# Faster Elbow MRI with Deep Learning Reconstruction—Assessment of Image Quality, Diagnostic Confidence, and Anatomy Visualization Compared to Standard Imaging

**DOI:** 10.3390/diagnostics13172747

**Published:** 2023-08-24

**Authors:** Judith Herrmann, Saif Afat, Sebastian Gassenmaier, Jan-Peter Grunz, Gregor Koerzdoerfer, Andreas Lingg, Haidara Almansour, Dominik Nickel, Theresa Sophie Patzer, Sebastian Werner

**Affiliations:** 1 Department of Diagnostic and Interventional Radiology, University Hospital Tübingen, 72076 Tübingen, Germanysebastian.gassenmaier@med.uni-tuebingen.de (S.G.); andreas.lingg@med.uni-tuebingen.de (A.L.); haidara.al-mansour@med.uni-tuebingen.de (H.A.); sebastian.werner@med.uni-tuebingen.de (S.W.); 2 Department of Diagnostic and Interventional Radiology, University Hospital Würzburg, 97080 Würzburg, Germany; grunz_j@ukw.de (J.-P.G.); patzer_t@ukw.de (T.S.P.); 3 MR Application Predevelopment, Siemens Healthcare GmbH, 91052 Erlangen, Germany; gregor.koerzdoerfer@siemens-healthineers.com (G.K.); marcel.nickel@siemens-healthineers.com (D.N.)

**Keywords:** acceleration, MRI, deep learning reconstruction, image processing, elbow

## Abstract

Objective: The objective of this study was to evaluate a deep learning (DL) reconstruction for turbo spin echo (TSE) sequences of the elbow regarding image quality and visualization of anatomy. Materials and Methods: Between October 2020 and June 2021, seventeen participants (eight patients, nine healthy subjects; mean age: 43 ± 16 (20–70) years, eight men) were prospectively included in this study. Each patient underwent two examinations: standard MRI, including TSE sequences reconstructed with a generalized autocalibrating partial parallel acquisition reconstruction (TSE_STD_), and prospectively undersampled TSE sequences reconstructed with a DL reconstruction (TSE_DL_). Two radiologists evaluated the images concerning image quality, noise, edge sharpness, artifacts, diagnostic confidence, and delineation of anatomical structures using a 5-point Likert scale, and rated the images concerning the detection of common pathologies. Results: Image quality was significantly improved in TSE_DL_ (mean 4.35, IQR 4–5) compared to TSE_STD_ (mean 3.76, IQR 3–4, *p* = 0.008). Moreover, TSE_DL_ showed decreased noise (mean 4.29, IQR 3.5–5) compared to TSE_STD_ (mean 3.35, IQR 3–4, *p* = 0.004). Ratings for delineation of anatomical structures, artifacts, edge sharpness, and diagnostic confidence did not differ significantly between TSE_DL_ and TSE_STD_ (*p* > 0.05). Inter-reader agreement was substantial to almost perfect (κ = 0.628–0.904). No difference was found concerning the detection of pathologies between the readers and between TSE_DL_ and TSE_STD_. Using DL, the acquisition time could be reduced by more than 35% compared to TSE_STD_. Conclusion: TSE_DL_ provided improved image quality and decreased noise while receiving equal ratings for edge sharpness, artifacts, delineation of anatomical structures, diagnostic confidence, and detection of pathologies compared to TSE_STD_. Providing more than a 35% reduction of acquisition time, TSE_DL_ may be clinically relevant for elbow imaging due to increased patient comfort and higher patient throughput.

## 1. Introduction

The elbow is a small but complex joint that can be affected by numerous traumatic, degenerative, and inflammatory pathologies. Its functional impairment can severely impact the quality of patients’ lives [1,2]. MRI is the optimal exam to assess the elbow joint’s cartilage, tendons, ligaments, and to a certain degree, its osseous components. As a standard non contrast-enhanced protocol, the German Radiological Society recommends axial and coronal fat-suppressed (fs) proton-density (PD)-weighted, coronal T1-weighted, sagittal PD- or T2-weighted and, as appropriate, axial PD- or T2-weighted sequences [3]. Our institution’s standard native examination lasts approximately 15 min. For optimal visualization of the elbow joint, the patient is preferably positioned prone with the arm elevated above their head in the so-called “Superman” position. Depending on the patient’s condition, maintaining this position for a long time can pose problems and lead to decreased image quality caused, for example, by motion artifacts. Therefore, a scan time reduction is desirable. Another aspect of MRI acceleration that is becoming increasingly important is the potential for reductions in energy consumption and, thus, an improved carbon footprint and reduced costs [4].

Adding to established methods such as compressed sensing and parallel imaging, recently, a relatively new form of MRI acceleration based on deep learning (DL) reconstruction has gained popularity. In image reconstruction, DL algorithms can be applied in various manners and differ regarding input and output: They can work purely on an image-to-image basis, for example, to decrease the amount of noise in an already reconstructed image. They can be used to directly reconstruct an image from the k-space data (“direct k-space to image mapping”). Furthermore, DL can be integrated into physics-based reconstruction techniques, either in the form of k-space learning, or iterative optimization within the image domain containing interleaved data consistency steps (unrolled optimization methods). One can even combine these latter two approaches into hybrid ones that learn a neural network in both the k-space and image domains [5].

The prototypical approach used in this study has been described in a different publication of our research group [6]. The technical foundations have been detailed in the publications by Hammernik et al. [7] and Schlemper et al. [8]. It combines deep learning-based reconstructions with conventional undersampling patterns as used in parallel imaging. These patterns are advantageous due to their clinical establishment, adaptability, and known artifact behavior. For image quality, calibration data for coil-sensitivity estimation are gathered. Specifically, during a TSE sequence, a region around the k-space center is fully sampled, helping both in image reconstruction and in estimating coil sensitivity maps. Our prototype image reconstruction uses an iterative reconstruction method or variational network. A notable feature of this method is the deep neural network model which combines the advantages of physical MR imaging models with those of data-driven ones. The fixed unrolled algorithm is made up of multiple cascades. The model’s architecture, which is more memory-efficient than traditional convolutional neural networks, is known as a “Deep, Iterative, Hierarchical Network”. Apart from the main undersampled k-space input data, coil-sensitivity maps and a bias field are also considered. When reconstructing images, data are fed into the variational network which also uses two new types of cascades: pre-cascades and post-cascades. The former concentrates on the parallel imaging aspect of the reconstruction issue, while the latter ensures data consistency. Lastly, the reconstruction model was trained on approximately 10,000 slices acquired from volunteers using conventional TSE protocols on 1.5 T and 3 T scanners. The data encompassed various contrasts, orientations, body regions, and resolutions. The input was retrospectively undersampled to an acceleration factor of 4 and the training was executed in PyTorch on an NVIDIA Tesla V100 GPU.

Several studies examining DL-based reconstruction techniques in the context of musculoskeletal imaging have already been published [6,7,8,9,10,11,12,13,14,15,16,17]. DL reconstruction can efficiently solve nonlinear and ill-posed reconstruction problems [18,19,20]. The training of such algorithms usually ensues in a supervised manner using representative, fully sampled data as a reference. The trained architecture can then be used prospectively to reconstruct an aliasing and noise-reduced image within a few seconds and with significantly reduced computational demand [14,21,22]. Multiple studies have demonstrated that this approach can improve image resolution, acquisition time, signal-to-noise ratio, and contrast-to-noise ratio while maintaining the original contrast [6,9,10,16,17].

We hypothesize that our prototypical DL-based reconstruction in elbow MRI will significantly shorten the examination time while maintaining high image quality and diagnostic confidence. Therefore, this study aims to evaluate the effectiveness of using DL to reconstruct turbo spin echo (TSE) images of the elbow and compare them to conventional TSE images. 

## 2. Materials and Methods

### 2.1. Study Design

The institutional review board approved this prospective single-center study. All procedures performed in studies involving human participants were in accordance with the ethical standards of the institutional research committee and with the 1964 Helsinki declaration and its later amendments. Participation in the study was voluntary, and informed consent was obtained from all individual participants included in the study. Siemens Healthcare, Erlangen, Germany, provided the prototype DL reconstruction application, but complete control of patient data was with the authors. Between October 2020 and June 2021, 17 participants were included, 9 healthy subjects and 8 consecutive patients with a clinical indication for elbow MRI. Each participant underwent a standard TSE protocol (TSE_STD_) and, in the same examination, an analogous protocol using the research DL reconstruction (TSE_DL_). The participants’ characteristics are displayed in Table 1.

### 2.2. Imaging Protocol

Participants were examined in clinically used 1.5 T and 3 T scanners (MAGNETOM Aera, MAGNETOM Avanto^fit^, MAGNETOM Skyra, MAGNETOM Prisma^fit^, and MAGNETOM Vida; Siemens Healthcare, Erlangen; Germany) using a 4-channel wrap-around extremity coil (4-channel Flex Coil, Siemens Healthcare, Erlangen; Germany). The institution’s standard protocol for imaging of the elbow consists of a native T1-weighted TSE in the coronal plane, a native proton density (PD)-weighted TSE with spectral fat saturation in the coronal, axial and sagittal plane, as well as optional native T1- and T2-weighted TSE in the sagittal plane. First, the institution’s standard protocol with TSE_STD_ using generalized autocalibrating partial parallel acquisition reconstruction (GRAPPA) was employed. Second, each TSE sequence was acquired a second time using deep learning reconstruction (TSE_DL_). Detailed acquisition parameters for TSE_STD_ and TSE_DL_ are given in Table 2. For TSE_DL_, the undersampling method and subsequent prototype DL reconstruction technique used in this study have already been described in detail in prior studies [7,8]. The k-space data are prospectively undersampled using established regular patterns from parallel imaging. K-space data, precomputed coil sensitivity maps, and a bias field for image homogenization are inserted into the variational network, consisting of multiple cascades.

The training of the DL reconstruction was conducted by Siemens Healthcare. It was performed on volunteer acquisitions using conventional TSE protocols independently of the data acquired in this study. About 10,000 slices were acquired in volunteers using clinical 1.5 T and 3 T scanners (MAGNETOM scanners, Siemens Healthcare, Erlangen, Germany). Using representative protocols for the respective body regions, fully sampled high-resolution acquisitions were performed in various anatomies, such as the head, pelvis, and knee. The training data included different image contrasts, orientations, body regions, and resolutions. The training was implemented in PyTorch and performed on a GPU cluster NVIDIA Tesla V100 (32 GB of memory) GPU.

For deployment in the clinical setting, the trained network was converted for prospective use in a proprietary C++ inference framework and integrated into the scanners’ reconstruction pipeline. 

### 2.3. Image Analysis

Image analysis was performed independently by two radiologists with seven years and three years of experience interpreting musculoskeletal MRI using a dedicated workstation (GE Healthcare Centricity™ PACS RA1000, Milwaukee, WI, USA). TSE_DL_ and TSE_STD_ sequences were separated, resulting in 34 datasets, and both readers were blinded toward reconstruction type, patient data, clinical and radiological reports, and each other’s assessments. Reading sessions were carried out in a random order consisting of datasets of both TSE_STD_ and TSE_DL_. 

Qualitative image analysis comprised the following items: image quality, artifacts, edge sharpness, noise, and diagnostic confidence. Additionally, we rated the quality of delineation of the following anatomical structures: radial collateral ligament (RCL), ulnar collateral ligament (UCL), lateral ulnar collateral ligament (LUCL), annular ligament, common flexor tendon, common extensor tendon, biceps tendon, brachialis tendon, triceps tendon, ulnar nerve, median nerve, radial nerve, and cartilage. Image quality items and delineation of anatomical structures were rated on an ordinal 5-point Likert scale (1 = non-diagnostic; 2 = poor; 3 = moderate; 4 = good; 5 = excellent).

Quantitative analysis comprised an evaluation of pathological lesions of the following anatomical structures: RCL, UCL, LUCL, annular ligament, common flexor tendon, common extensor tendon, biceps tendon, brachialis tendon, triceps tendon, ulnar nerve, median nerve, radial nerve, and cartilage (0 = absent; 1 = present).

Furthermore, quantitative analysis of SNR was performed in all patient datasets via measurement of the signal intensities (SI) and standard deviation using the following formula:

$$\mathrm{SNR}=\frac{SI}{\left(standard\;deviation\right)}$$

A region of interest was manually drawn in the exact same location on TSE_STD_ and TSE_DL_ in the brachialis muscle and the capitulum of the humerus on axial slices of the PD-weighted TSE sequences. Large vessels and focal lesions were avoided for measurements.

### 2.4. Statistical Analysis

Descriptive statistics were used to summarize participants’ demographics. The mean, median, and interquartile range are reported for ordered categorical variables, and the mean and standard deviation for continuous variables. A paired-sample Wilcoxon signed-rank test was used to compare the sequences concerning image quality by each reader. Inter-reader agreement was used to assess weighted Cohen’s κ, both with 95% confidence intervals, and interpreted as follows: 0.20 or less, poor agreement; 0.21–0.40, fair agreement; 0.41–0.60, moderate agreement; 0.61–0.80, substantial agreement; and greater than 0.80, almost perfect agreement. Significance was set at a level of *p* < 0.05. Statistical analyses were performed using SPSS version 26 (IBM Corp, Armonk, NY, USA).

## 3. Results

**Figure 1:**
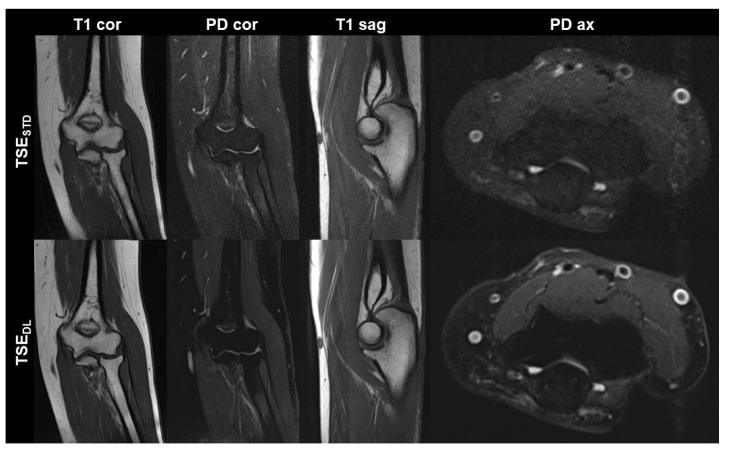
Non-contrast elbow MRI acquired at 1.5 T in a 51-year-old female patient with the institution’s standard turbo spin echo (TSE) sequences (TSE_STD_, **upper row**) and deep learning-reconstructed TSE sequences (TSE_DL_, **lower row**). Increased edge sharpness in the TSE_DL_ images is primarily seen in the coronal and sagittal T1-weighted sequences (T1 cor and T1 sag). In the fat-saturated coronal and axial PD-weighted images (PD cor and PD ax) the DL reconstruction shows decreased noise.

**Figure 2:**
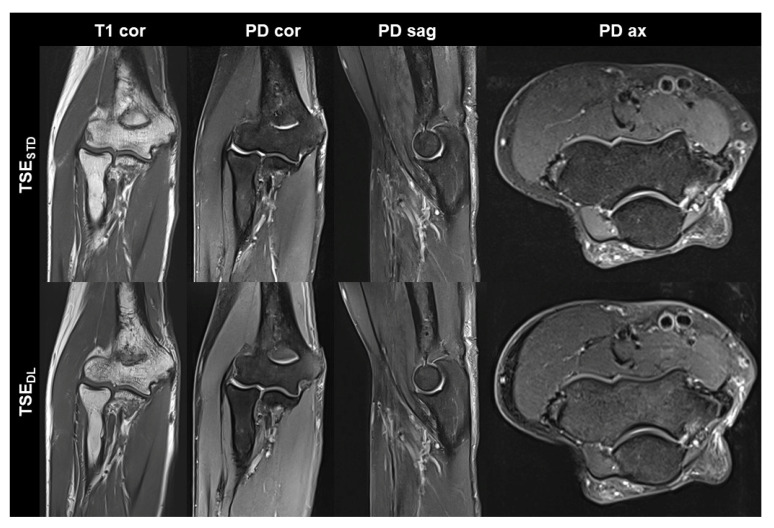
Non-contrast elbow MRI acquired at 3 T in a 70-year-old male patient with the institution’s standard turbo spin echo (TSE) sequences (TSE_STD_, **upper row**) and deep learning-reconstructed TSE sequences (TSE_DL_, **lower row**). In this example the standard and DL-reconstructed images show very similar image quality. In the TSE_DL_ images, all anatomic details are well depicted, showing that the reduced acquisition time did not lead to a loss in detail. Additionally, edge sharpness in the coronal T1-weighted images (T1 cor) is slightly increased in the TSE_DL_ image.

**Figure 3:**
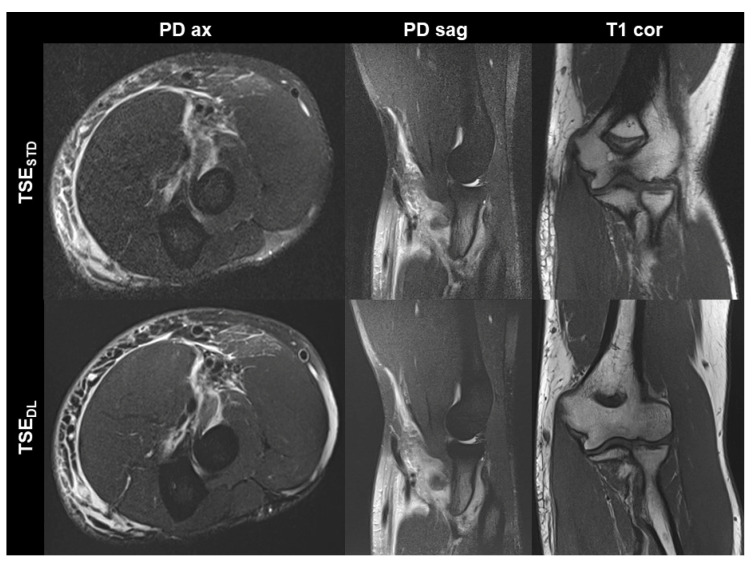
Elbow MRI acquired at 1.5 T in a 36-year-old male patient with clinically suspected biceps tendon tear after trauma with the institution’s standard turbo spin echo (TSE) sequences (TSE_STD_, **upper row**) and deep learning-reconstructed TSE sequences (TSE_DL_, **lower row**). The MRI examination confirmed the suspicion of a partial tear of the biceps’ tendon at its insertion with a peritendinous hematoma. Noise is reduced in the DL-reconstructed axial and sagittal fat-suppressed PD-weighted images (PD ax and PD sag). The intact ulnar collateral ligament is depicted well in the standard and DL-reconstructed coronal T1-weighted images; the difference in angulation between the two images is due to movement between acquisitions.

**Figure 4:**
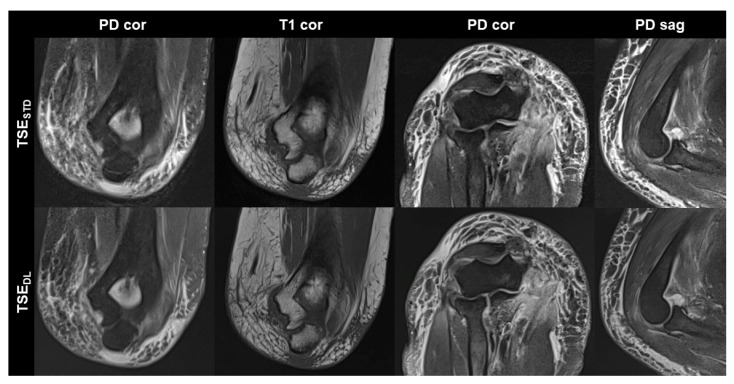
Elbow MRI acquired at 1.5 T with the institution’s standard turbo spin echo (TSE) sequences (TSE_STD_, **upper row**) and deep learning reconstructed TSE sequences (TSE_DL_, **lower row**) in a 66-year-old female patient who had suffered an elbow dislocation. The patient could not extend the elbow for the examination. The images show an avulsion of the common flexor tendon with hemorrhage into the flexor muscles (PD cor and PD cor), a complete tear of the radial collateral ligament (PD cor, third column) and a dislocated annular ligament (PD sag). The fat-supressed PD-weighted TSE_DL_ images show improved noise, while the DL-reconstructed coronal T1-weighted image shows very similar image quality compared to the standard image.

TSE_DL_ was successfully performed in each of the 17 included participants (mean age 43 ± 16 (20–70), years, eight men). Seven exams were performed at 1.5 T and 10 exams at 3 T. TSE_DL_ enabled a scan time reduction by more than 35% at 1.5 T (TSE_DL_ 8:19 min vs. TSE_STD_ 13:06 min) and by more than 55% at 3 T (TSE_DL_ 6:48 min vs. TSE_STD_ 15:15 min). Image examples with comparisons of TSE_STD_ and TSE_DL_ are displayed in Figure 1, Figure 2, Figure 3 and Figure 4.

The inter-reader agreement was substantial to almost perfect, with values between 0.648 and 0.946. Therefore, only the results of the more experienced reader 1 are given hereafter. A summary of all qualitative image analyses is provided in Table 3.

The overall image quality of TSE_DL_ (4.35, median 4, IQR 4–5) was rated superior to TSE_STD_ (3.76, median 4, IQR 3–4, *p* = 0.008). In addition, TSE_DL_ noise levels received significantly higher ratings, i.e., lower noise (4.29, median 5, IQR 3.5–5) than TSE_STD_ (3.35, median 3, IQR 3–4, *p* = 0.004).

We found no significant differences between TSE_DL_ and TSE_STD_ regarding edge sharpness (TSE_DL_: 4.12, median 4, IQR 4–5; TSE_STD_: 3.94, median 4, IQR 4–4, *p* = 0.257), the extent of artifacts (TSE_DL_: 4.24, median 4, IQR 4–4.5; TSE_STD_: 4.06, median 4, IQR 4–4, *p* = 0.083) and diagnostic confidence (TSE_DL_: 4.47, median 4, IQR 4–5; TSE_STD_: 4.47, median 4, IQR 4–5, *p* = 0.180).

No significant difference was found concerning the delineation of anatomical structures between TSE_DL_ and TSE_STD_ (*p* > 0.05; see Table 3). 

The quantitative analysis revealed no difference between the sequences as well as the readers concerning the detection of pathologies in the most important anatomical structures as an equal number of pathological lesions was detected between both readers as well as between TSE_STD_ and TSE_DL_ (RCL, number (*n*) = 4/17; UCL, *n* = 1/17; LUCL, *n* = 2/17; annular ligament, *n* = 1/17; common flexor tendon, *n* = 1/17; common extensor tendon, *n* = 4/17; biceps tendon, *n* = 5/17; brachialis tendon, *n* = 3/17; triceps tendon, *n* = 0/17; ulnar nerve, *n* = 0/17; median nerve, *n* = 0/17; radial nerve, *n* = 0/17; and cartilage *n* = 4/17). 

The measurement of SNR resulted in significantly higher values for TSE_DL_ versus TSE_STD_ for both tissues (muscle: TSE_DL_ 9.06 ± 2.67, TSE_STD_ 6.72 ± 2.11, *p* = 0.012; bone: TSE_DL_ 8.72 ± 2.88, TSE_STD_ 6.80 ± 4.19, *p* = 0.036). 

## 4. Discussion

Our study examined the practicality of using a deep learning-based reconstruction method for prospectively undersampled TSE images of the elbow. To our knowledge, this is the first time this approach has been explicitly studied for elbow MRI. The results indicate that a more than 35% reduction in scan time is possible compared to conventional TSE images. This is accomplished while maintaining similar edge sharpness, artifacts, anatomy delineation quality, confidence in the diagnosis, and detection of common pathologies. Additionally, the deep learning-based method improves overall image quality and decreases noise.

The time-saving aspect of our approach can eventually be beneficial to either patient comfort or to a higher throughput. We were able to achieve a scan time reduction from approximately 15 min to 7–8 min for both field strengths. We currently schedule an elbow MRI for a 30 min time slot including preparing the examination and elbow positioning, which can be challenging and time-consuming, but often does not necessitate 15 min. Thus, the reduction of scan time to 7–8 min might possibly allow for scheduling an elbow MRI within a 20 min time slot. This would mean increasing the number of examinations by a factor of 1.5. However, an aspect that has yet to receive much attention, but is becoming increasingly important, is the potential for savings in energy consumption [4]. To what extent, besides the energy savings due to shortened scan times, DL-based reconstruction techniques lead to lower energy consumption per time unit has yet to be discovered. Regarding this question, one also has to account for increased energy consumption during the DL algorithm training process. Members of our research group are currently investigating this question.

To date, no published study has addressed elbow MRI specifically regarding scan-time reduction using DL-based or non-DL-based methods. Six elbow MRIs were included in a study by Delattre et al. [23] investigating the time-saving effects of compressed sensing in multiple anatomic regions. The sequence they used was a 3D TSE PD-weighted volume isotropic turbo spin echo sequence with spectral attenuated inversion recovery fat suppression. They reported a 33% reduction in scan time for elbow MRI, albeit the elbow being the only anatomic region in which the application of compressed sensing significantly lowered the CNR. For wrist and knee MRI, they achieved a scan time reduction of 32% and 33%, respectively, without a significant noise increase. A recently published study by Johnson et al. examined DL-based reconstruction in 3 T knee MRIs of 170 patients and compared them to conventional sequences using a generalized autocalibrating partial parallel acquisition. They reported a time reduction of approximately 45% (from approximately 10 min to 5.5 min) while achieving clinical interchangeability and improved overall image quality, sharpness, and signal-to-noise scores. Kim et al. [9], with the combined use of DL-enhanced eight-fold acceleration imaging (four-fold parallel imaging with two-fold simultaneous multi-slice imaging) were able to achieve similar performance compared to conventional two-fold parallel imaging for assessing pathologies in 33 knee MRIs, reducing scan time by 71%. Foreman et al. [10] prospectively evaluated 30 3 T ankle MRIs comparing DL-enhanced compressed sensing with conventional compressed sensing reconstruction. The study showed that DL reconstruction reduced acquisition times by 47% without losses in diagnostic image quality. Furthermore, they showed that the technique could alternatively be used to achieve higher image resolution when foregoing the reduction in acquisition time.

Certain limitations of our findings should be considered when interpreting them. These limitations include the small sample size and the fact that the study was conducted at a single center, which may affect the generalizability of the results. Additionally, the parameters used to acquire the images were not entirely consistent between the different scanners, and we included healthy volunteers and patients in the study. Although the readers were unaware of which reconstruction technique was used, characteristic differences in the images could have influenced the results. Furthermore, all scanners used in the study were from the same manufacturer. Future research should involve a larger patient population and more standardized protocols, and investigate whether DL-based reconstruction can improve diagnostic accuracy when acceleration is not used.

To conclude, our technique using deep learning for reconstruction resulted in a more than 35% reduction in acquisition time while maintaining an accurate representation of anatomy, diagnostic confidence, edge sharpness, and detection of pathologies. Additionally, our approach significantly improved noise reduction and overall image quality. These results have potential clinical implications for elbow imaging, possibly resulting in increased patient comfort and efficiency by significantly reducing the total scan time. In addition, in the current era, it is also essential to keep in mind that a shortened scan time has the potential for reduced energy consumption, although to what extent this is really the case remains to be seen and is an interesting question for future studies.

## Figures and Tables

**Table 1:** Participants’ characteristics.

Characteristics	Values
Total (male/female), *n*	17 (8/9)
Patients/healthy subjects, *n*	8/9
Age, mean ± SD (range), y	total: 43 ± 16 (20–70)
	male: 48 ± 14 (27–70)
	female: 38 ± 16 (20–66)
Scanner (1.5/3 T), *n*	7/10
Indication of MRI in patients, *n*	Posttraumatic, 3
	Epicondylitis, 2
	Arthritis/Enthesitis, 2
	Other, 1

SD, standard deviation; y, years; *n*, number.

**Table 2:** Example MRI acquisition parameters.

Tesla	Sequence	Orientation	FS	TA	FOV	Matrix	Res.	ST	Slices	TE	TR	ETL	FA	PI	AV	PB
3	PD TSE_STD_	coronal	FS	2:58	180 × 180	320 × 320	0.56	3	24	46	3720	9	134	0	1	200
	PD TSE_DL_	coronal	FS	1:12	180 × 180	320 × 320	0.56	3	24	37	3000	9	120	2	1	200
	PD TSE_STD_	axial	FS	4:30	180 × 180	320 × 320	0.56	3	32	45	3000	7	120	2	1	180
	PD TSE_DL_	axial	FS	2:01	180 × 180	320 × 320	0.56	3	32	51	5040	7	120	2	1	180
	PD TSE_STD_	sagittal	FS	4:19	180 × 180	384 × 384	0.46	3	24	47	3820	9	134	0	1	200
	PD TSE_DL_	sagittal	FS	1:47	180 × 180	384 × 384	0.46	3	24	47	3160	9	150	2		200
	T1 TSE_STD_	coronal	none	3:28	180 × 180	384 × 384	0.46	3	24	12	598	3	150	0	1	160
	T1 TSE_DL_	coronal	none	1:48	180 × 180	384 × 384	0.46	3	24	12	598	3	160	2	1	160
1.5	PD TSE_STD_	coronal	FS	2:24	180 × 180	320 × 320	0.56	3	20	50	3000	9	134	0	1	200
	PD TSE_DL_	coronal	FS	0:51	180 × 180	320 × 320	0.56	3	20	50	3000	13	134	2	1	200
	PD TSE_STD_	axial	FS	4:00	180 × 180	320 × 320	0.56	3	34	43	3000	7	150	2	1	180
	PD TSE_DL_	axial	FS	1:57	180 × 180	320 × 320	0.56	3	34	47	5120	13	150	2	1	180
	PD TSE_STD_	sagittal	FS	4:00	180 × 180	384 × 384	0.46	3	24	52	3540	9	134	0	1	200
	PD TSE_DL_	sagittal	FS	1:18	180 × 180	384 × 384	0.46	3	24	52	3410	13	134	2	1	200
	T1 TSE_STD_	coronal	none	2:42	180 × 180	384 × 384	0.46	3	20	11	467	3	150	0	1	160
	T1 TSE_DL_	coronal	none	1:13	180 × 180	384 × 384	0.46	3	20	11	580	4	150	2	1	160
	T2 TSE_STD_	sagittal	none	3:23	180 × 180	384 × 384	0.46	3	26	72	4720	14	150	0	1	150
	T2 TSE_DL_	sagittal	none	1:28	180 × 180	384 × 384	0.46	3	26	115	3860	13	150	2	1	150
	T1 TSE_STD_	sagittal	none	2:54	180 × 180	384 × 384	0.46	3	24	11	505	3	150	2	1	160
	T1 TSE_DL_	sagittal	none	1:28	180 × 180	384 × 384	0.46	3	24	11	464	4	150	3	1	160

TSE_STD_, standard TSE; TSE_DL_, deep learning-reconstructed TSE; PD, proton density; TSE, turbo spin echo; DL, deep learning; FS, fat saturation; TA, time of acquisition (min); FOV, field of view (mm); Res., in-plane resolution (mm); ST, slice thickness; slice thickness (mm); TE/TR, echo time/repetition time (ms); ETL, echo train length; FA, flip angle (degree); PI, parallel imaging factor; AV, averages; PB, pixel bandwidth. There are no parameters listed for the optional sagittal T1- and T2-weighted sequences at 3 T because there were no cases in which these sequences were acquired at 3 T.

**Table 3:** Image quality and inter-reader agreement of standard TSE (TSE_STD_) and deep learning-reconstructed TSE (TSE_DL_).

Item	Reader 1			Reader 2			Cohen’s κ	
	TSE_STD_	TSE_DL_	*p*-Value	TSE_STD_	TSE_DL_	*p*-Value	TSE_STD_	TSE_DL_
Image quality	3.76 (4; 3–4)	4.35 (4; 4–5)	0.008	3.82 (4; 3.5–4)	4.29 (4, 5–5)	0.011	0.883	0.749
Noise	3.35 (3; 3–4)	4.29 (5; 3.5–5)	0.004	3.41 (4; 3–4)	4.47 (5; 4–5)	0.001	0.919	0.777
Edge sharpness	3.94 (4; 4–4)	4.12 (4; 4–5)	0.257	3.94 (4; 3.5–4)	4.24 (4; 4–4)	0.096	0.798	0.813
Artifacts	4.06 (4; 4–4)	4.24 (4; 4–4.5)	0.083	4.06 (4; 4–4)	4.18 (4; 4–4)	0.317	0.717	0.821
Diagnostic confidence	4.47 (4; 4–5)	4.65 (5; 4–5)	0.180	4.53 (5; 4–5)	4.65 (5; 4–5)	0.157	0.648	0.742
RCL	4.65 (5; 4–5)	4.59 (5; 4–5)	0.564	4.53 (5; 4–5)	4.71 (5; 4–5)	0.083	0.779	0.746
UCL	4.65 (5; 4–5)	4.59 (5; 4–5)	0.655	4.71 (5; 4–5)	4.59 (5; 4–5)	0.317	0.866	0.793
LUCL	4.12 (4; 4–5)	4.41 (4; 4–5)	0.059	4.18 (4; 4–5)	4.47 (4; 4–5)	0.096	0.919	0.881
Anular ligament	4.47 (5; 4–5)	4.65 (5; 4–5)	0.083	4.59 (5; 4–5)	4.65 (5; 4–5)	0.564	0.805	0.776
Flexors	4.65 (5; 4–5)	4.47 (5; 4–5)	0.366	4.65 (5; 3–5)	4.59 (5; 4–5)	0.705	0.764	0.787
Extensors	4.35 (4; 4–5)	4.65 (5; 4–5)	0.096	4.41 (4; 4–4)	4.53 (5; 4–5)	0.480	0.733	0.779
Biceps	4.47 (5; 4–5)	4.59 (5; 4–5)	0.317	4.53 (5; 4–5)	4.47 (5; 4–5)	0.564	0.903	0.609
Triceps	4.29 (4; 4–5)	4.53 (5; 4–5)	0.234	4.41 (5; 4–5)	4.47 (5; 4–5)	0.748	0.857	0.894
Ulnaris	4.00 (4; 3–4)	4.24 (4; 3.5–4)	0.206	4.00 (4; 3–5)	4.29 (5; 3.5–5)	0.166	0.871	0.795
Medianus	3.76 (4; 3–4.5)	3.76 (4; 3–4.5)	0.763	3.71 (4; 3–4.5)	3.94 (4; 3–5)	0.366	0.946	0.825
Radialis	3.65 (4; 3–4)	3.71 (4; 3–4)	0.564	3.53 (4; 3–4)	3.71 (4; 3–4)	0.083	0.870	0.830
Brachialis	4.53 (5; 4–5)	4.65 (5; 4–5)	0.317	4.53 (5; 4–5)	4.59 (5; 4–5)	0.655	0.802	0.628
Cartilage	3.94 (4; 3.5–4)	4.00 (4; 4–4)	0.655	3.88 (4; 3.5–4)	4.00 (4; 4–4)	0.414	0.904	0.747

RCL, radial collateral ligament; UCL, ulnar collateral ligament; LUCL, lateral ulnar collateral ligament; Cohen’s κ, inter-reader agreement between the two readers.

## Data Availability

The datasets used and analyzed during the current study are available from the corresponding author on reasonable request.
